# Supplementary material for: Separability and geometry of object manifolds in deep neural networks
Source: Nat Commun. 2020 Feb 6;11:746. doi: 10.1038/s41467-020-14578-5 (PMC7005295; doi:10.1038/s41467-020-14578-5)
Supplement: Supplementary file 1 — Supplementary Information [file 41467_2020_14578_MOESM1_ESM.pdf]

Supplementary Information for "Separability and Geometry of Object Manifolds in Deep Neural Networks"

December 22, 2019

Contents

1 Figures **2**

1.1 Object manifolds in deep convolutional networks (AlexNet, VGG-16, ResNet-50) 2

1.1.1 Capacity for point-cloud manifolds in AlexNet, VGG-16, ResNet-50 2

1.1.2 Capacity for smooth manifolds in AlexNet, VGG-16, ResNet-50 3

1.1.3 Geometry of point-cloud and smooth manifolds in AlexNet, VGG-16, ResNet-50 5

1.1.4 Geometry of 1-d vs 2-d smooth manifolds in AlexNet, VGG-16, ResNet-50 8

1.1.5 Manifold correlations and their effect on capacity in AlexNet, VGG-16, ResNet-50 9

1.1.6 Deep network building-blocks have a consistent effect on manifold geometry and correlations 11

1.2 Predictions of manifold separability theory 13

1.2.1 Comparison between theory and numerically measured capacity in smooth manifolds of AlexNet, VGG-16, ResNet-50 13

1.2.2 Manifold capacity's dependence on number of objects and neurons 13

1.2.3 Comparison between full theory and approximation with geometric properties for capacity of smooth manifolds of AlexNet, VGG-16, ResNet-50 15

1.2.4 Random sampling versus random projection 16

2 Methods **18**

2.1 Measuring capacity and geometric manifold properties 18

2.2 Measuring manifold capacity numerically 20

2.3 ImageNet classes used for point-cloud manifolds 21

3 Notes **22**

3.1 Theory for low-rank center correlations 22

3.2 Theory for manifolds of random point 24

1 Figures

1.1 Object manifolds in deep convolutional networks (AlexNet, VGG-16, ResNet-50)

In this section we demonstrate that the overall trend of the improvement in capacity, reduction in dimension and radius across layers of a deep network generalizes to additional data-sets. Those include additional manifolds and another network model, ResNet-50. We show the results on AlexNet, VGG-16 alongside ResNet-50 for comparison.

1.1.1 Capacity for point-cloud manifolds in AlexNet, VGG-16, ResNet-50

The capacity of point-cloud manifolds created from ImageNet classes increases across the layers of ResNet-50 (figure 1). Furthermore, the capacity measured in AlexNet, VGG-16, ResNet-50 exhibit the same trend as their respective performance in the ImageNet object classification task for which they were trained. This trend continues when using different residual networks of different depths (figure 2a), where most of the capacity improvement is achieved in the final layers.

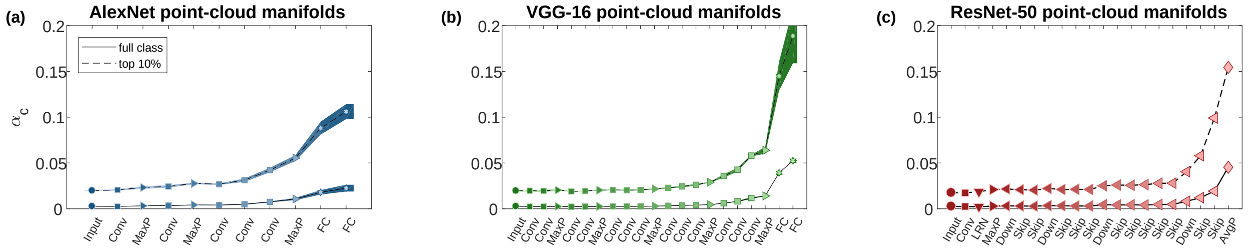

Figure 1: Capacity for point-cloud manifolds for ImageNet classes. Classification capacity for point-cloud manifolds (full line: full class manifolds; dashed line: top 10% manifolds) along the layers of deep neural networks: AlexNet (a) VGG-16 (b) and ResNet-50 (c). Line and markers at (a,b) indicate mean value over 4 choices of 64 objects and surrounding shaded areas indicate 95% confidence interval, while for (c) only the first set of objects is used.
The x-axis labels provides abbreviation of the layer types ('Input'- pixel layer, 'Conv'- convolutional layer, 'MaxP'- max-pooling layer, 'FC'- fully connected layer, 'AveP'- average pooling layer, 'LRN'- local normalization layer, 'Skip'- skip module, 'Down'- skip module with downsampling). Marker shape represents layer type (circle- pixel layer, square- convolution layer, right-triangle- max-pooling layer, hexagon- fully connected layer, diamond- average pooling layer, down-triangle- local normalization layer, left-triangle- a skip module). Color (blue- AlexNet, green- VGG-16, red- ResNet-50) changes from dark to light along the network. Features in linear layers ('Conv', 'FC') and skip modules ('Skip', 'Down') are extracted after a ReLU non-linearity.

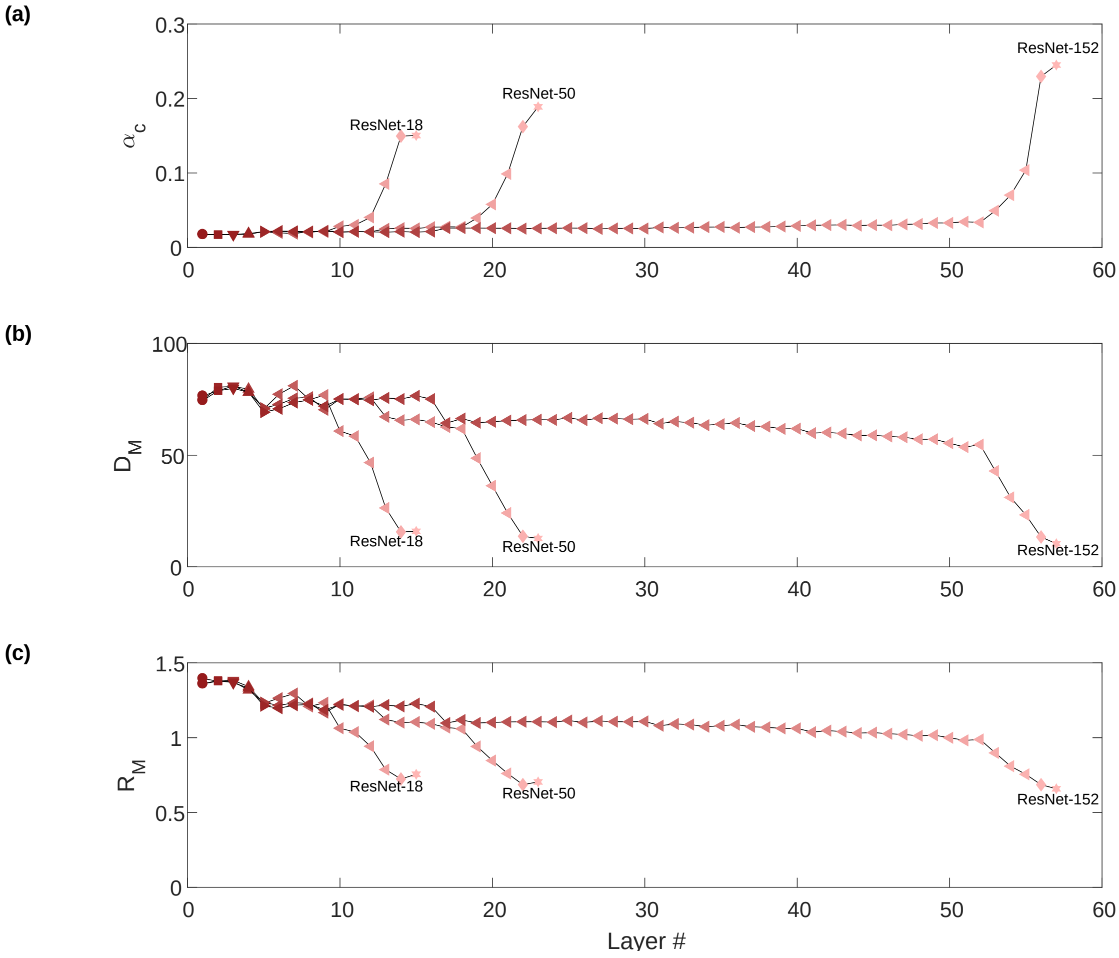

Figure 2: Capacity and geometry for point-cloud manifolds using residual networks. Classification capacity (a) mean manifold dimensions (b) and mean manifold radii (c) for point-cloud top 10% manifolds along the layers of deep residual networks (ResNet-18, ResNet-50, ResNet-152).
ResNet-50 results are the same as the first set of objects used for the previously presented results in supplementary figure 1. The x-axis indicate layer depth starting from the pixel layer. Marker shape represents layer type (circle- pixel layer, square- convolution layer, right-triangle- max-pooling layer, hexagon- fully connected layer, diamond- average pooling layer, down-triangle- local normalization layer, left-triangle- a skip module). Features in linear layers and skip modules are extracted after a ReLU non-linearity.

1.1.2 Capacity for smooth manifolds in AlexNet, VGG-16, ResNet-50

When calculating capacity for smooth manifolds a finite number of samples is used; figure 3 justify this practice by showing that for both 1-d and 2-d smooth manifolds when the number of samples tend to infinity capacity is still finite, and is very closed to the values measured using the maximal number of samples.

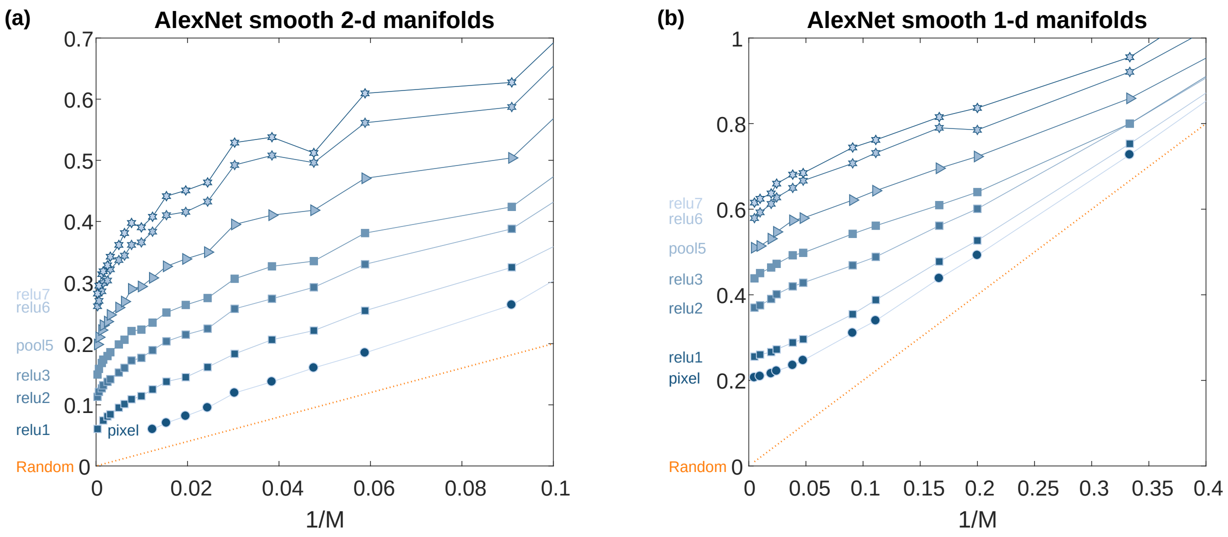

Figure 3: Capacity dependence on the number of samples in smooth manifolds.
(a-b) Numerically measured capacity for smooth manifolds across layers of AlexNet at using different number of samples (M) per manifold. Orange dashed line indicate the capacity expected for random points (2/M, see Methods).
(a) capacity (y-axis) vs inverse the number of samples (x-axis) for 2-d smooth manifolds.
(b) capacity (y-axis) vs inverse the number of samples (x-axis) for 1-d smooth manifolds.
Marker shape represents layer type (circle- pixel layer, square- convolution layer, right-triangle- max-pooling layer, hexagon- fully connected layer, diamond- average pooling layer, down-triangle- local normalization layer, left-triangle- a skip module). Color changes from dark to light along the network.

The capacity of smooth manifolds created from warped ImageNet images increases across the layers of ResNet-50 (figure 4), as demonstrated for AlexNet and VGG-16 at main figure 5. Furthermore, the capacity improvement achieved increases supra-linearly with the complexity of the manifold, quantified by its total variability at the pixel layer. Notably, using the theoretical relation between capacity and manifold properties (demonstrated empirically at main figure 10) most of the observed changes in capacity can be attributed to changes in dimensionality while changes in radii contribute just in the final layers (figure 7).

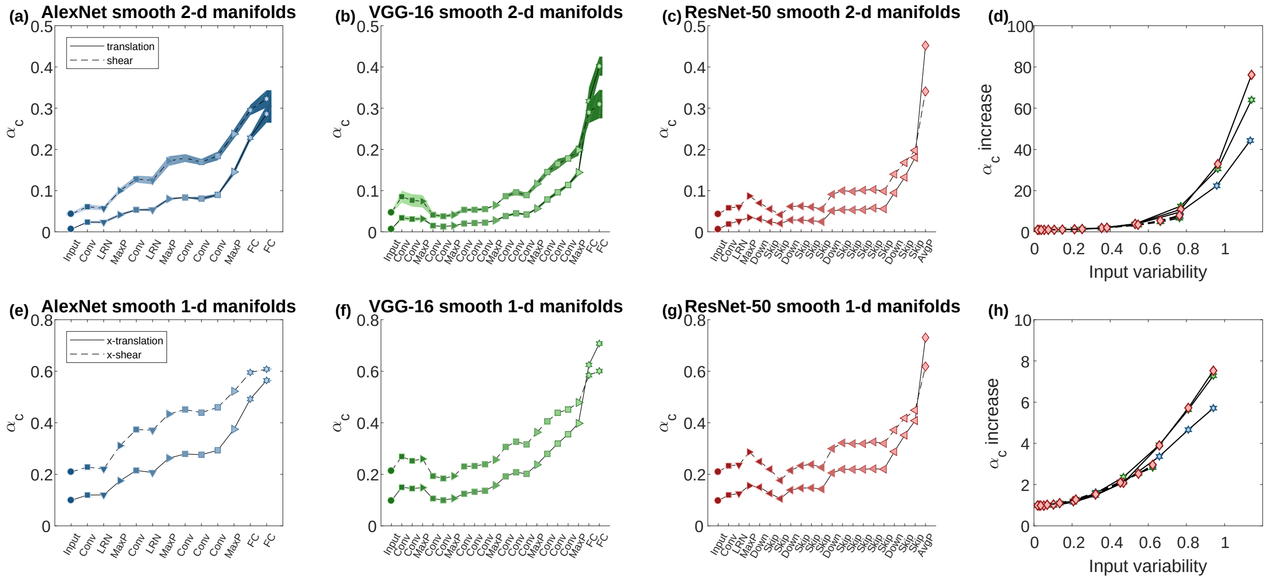

Figure 4: Capacity for smooth manifolds from warped ImageNet images. (a-c) Classification capacity for smooth manifolds (full line: translation; dashed line: shear) along the layers of AlexNet (a,e), VGG-16 (b,f) and ResNet-50 (c,g). The x-axis labels provides abbreviation of the layer types. Line and markers at (a,b) indicate mean value over different choices of objects and surrounding shaded areas at indicate 95% confidence interval, while at other panels only the first set of objects used. Marker shape represents layer type (circle- pixel layer, square- convolution layer, right-triangle- max-pooling layer, hexagon- fully connected layer, diamond- average pooling layer, down-triangle- local normalization layer, left-triangle- a skip module). Features in linear layers are extracted after a ReLU non-linearity. Color (blue- AlexNet, green- VGG-16, red- ResNet-50) changes from dark to light along the network.
(d,h) Capacity increase from the input (pixel layer) to the output (features layer) of AlexNet (blue markers), VGG-16 (green markers) and ResNet-50 (red markers) for smooth manifolds created from 2-d translation (d) or 1-d translation (h). The capacity increase is specified as ratio of capacity at the output layer relative to the pixel layer (y-axis), at different levels of stimuli variation, measured using SI equation (3) at the pixel layer (x-axis).

1.1.3 Geometry of point-cloud and smooth manifolds in AlexNet, VGG-16, ResNet-50

The geometry of point-cloud and smooth manifolds is characterized by the manifolds' dimensions and radii.

The mean values of manifold dimensions across the layers of AlexNet, VGG-16 and ResNet-50 are shown in figure 5, along with 95% confidence intervals with respect to sampling of different object. This serve to demonstrate a large but non-monotonic reduction in dimension along each hierarchy. Going beyond the mean, the distribution of dimension values is shown for selected manifolds in 5g-i. The manifold dimension D_M is comparable in magnitude and layer-by-layer changes to a well-known measure of dimension, the spectral participation ratio $D_{svd} = (\Sigma_i \lambda_i^2)^2/(\Sigma_i \lambda_i^4)$ where $\{\lambda_i\}_{i=1}^M$ are the manifold spectrum (i.e. singular values of centered manifold samples), shown for comparison at 5j-l.

Similarly, the mean values of manifold radii along the layers of AlexNet, VGG-16 and ResNet-50 are shown in figure 6, demonstrating a small and monotonic reduction in radius along each hierarchy for point-cloud manifolds while for smooth manifolds the main decrease is in the first layer and last layers (see discussion in main text). Going beyond the mean, the distribution of radii values is shown for selected manifolds in 6g-i.

For residual networks of different depths, we find that the imprved capacity improvement in final layers is associated with decrease f both radius and dimension (figure 2b-c).

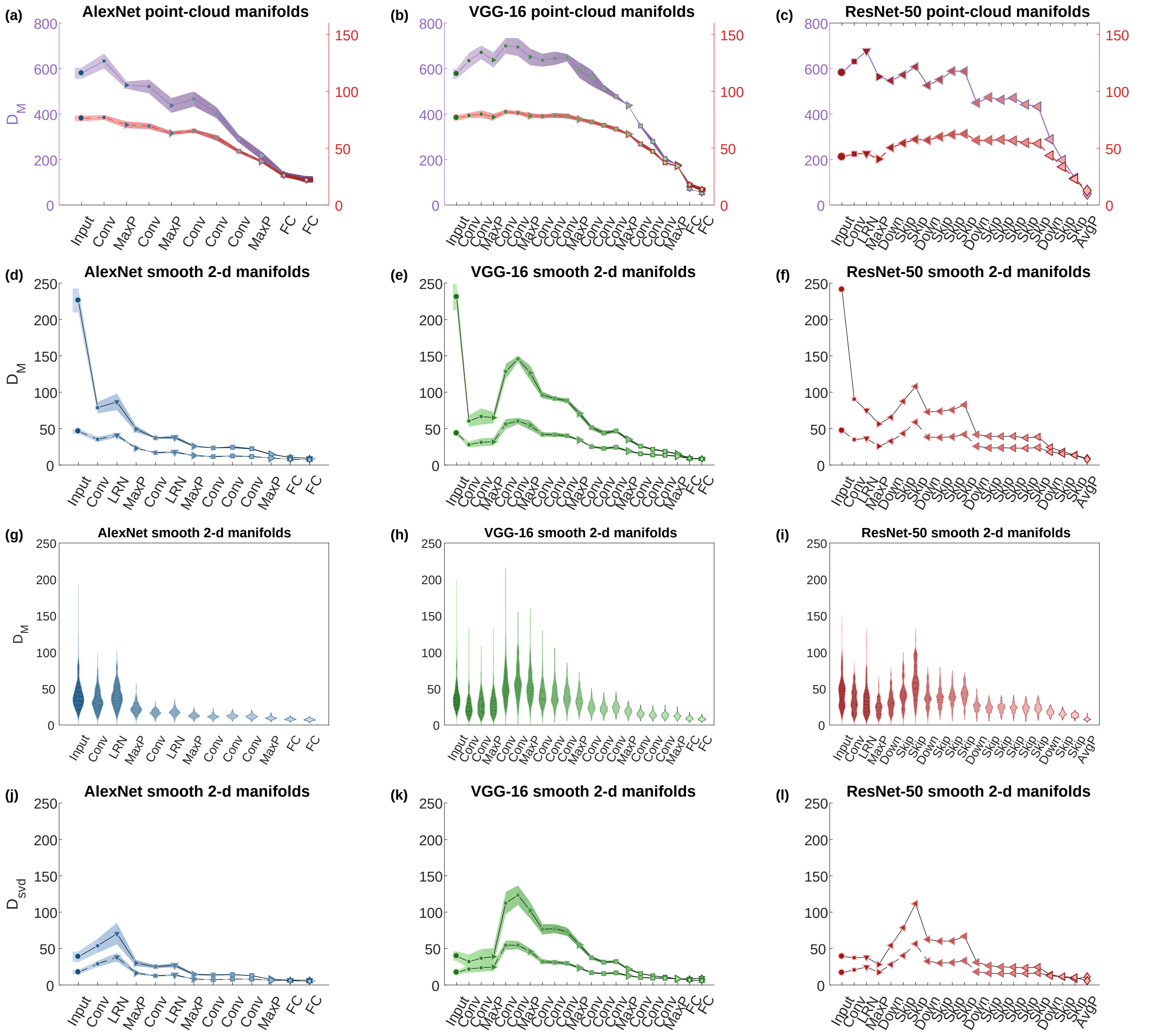

Figure 5: Manifold dimensions along the hierarchy.
(a-c) Mean manifold dimension for point-cloud manifolds of AlexNet (a), VGG-16 (b) and ResNet-50 (c); full-line: full-class manifolds, dashed-line: top 10% manifolds. Values of top 10% manifolds are showed against a secondary y-axis (color-coded by the markers edge) to improve visibility. (d-f) Mean manifold dimension for smooth 2-d manifolds of AlexNet (d), VGG-16 (e) and ResNet-50 (f); full-line: translation manifolds, dashed-line: shear manifolds. (g-i) Distribution of manifold dimension for smooth 2-d shear manifolds for the same deep networks as (d-f), illustrated as per-layer histogram (kernel width is std/5). (j-l) Mean manifold participation ratio for smooth 2-d manifolds of AlexNet (j), VGG-16 (k) and ResNet-50 (l); full-line: translation manifolds, dashed-line: shear manifolds.
Line and markers at (a,b,d,e,j,k) indicate mean value over different choices of objects and surrounding shaded areas at indicate 95% confidence interval, while at (c,f,l) only the first set of objects used. The x-axis labels provides abbreviation of the layer types. Marker shape represents layer type (circle- pixel layer, square- convolution layer, right-triangle- max-pooling layer, hexagon- fully connected layer, diamond- average pooling layer, down-triangle- local normalization layer, left-triangle- a skip module). Features in linear layers are extracted after a ReLU non-linearity. Color (blue- AlexNet, green- VGG-16, red- ResNet-50) changes from dark to light along the network.

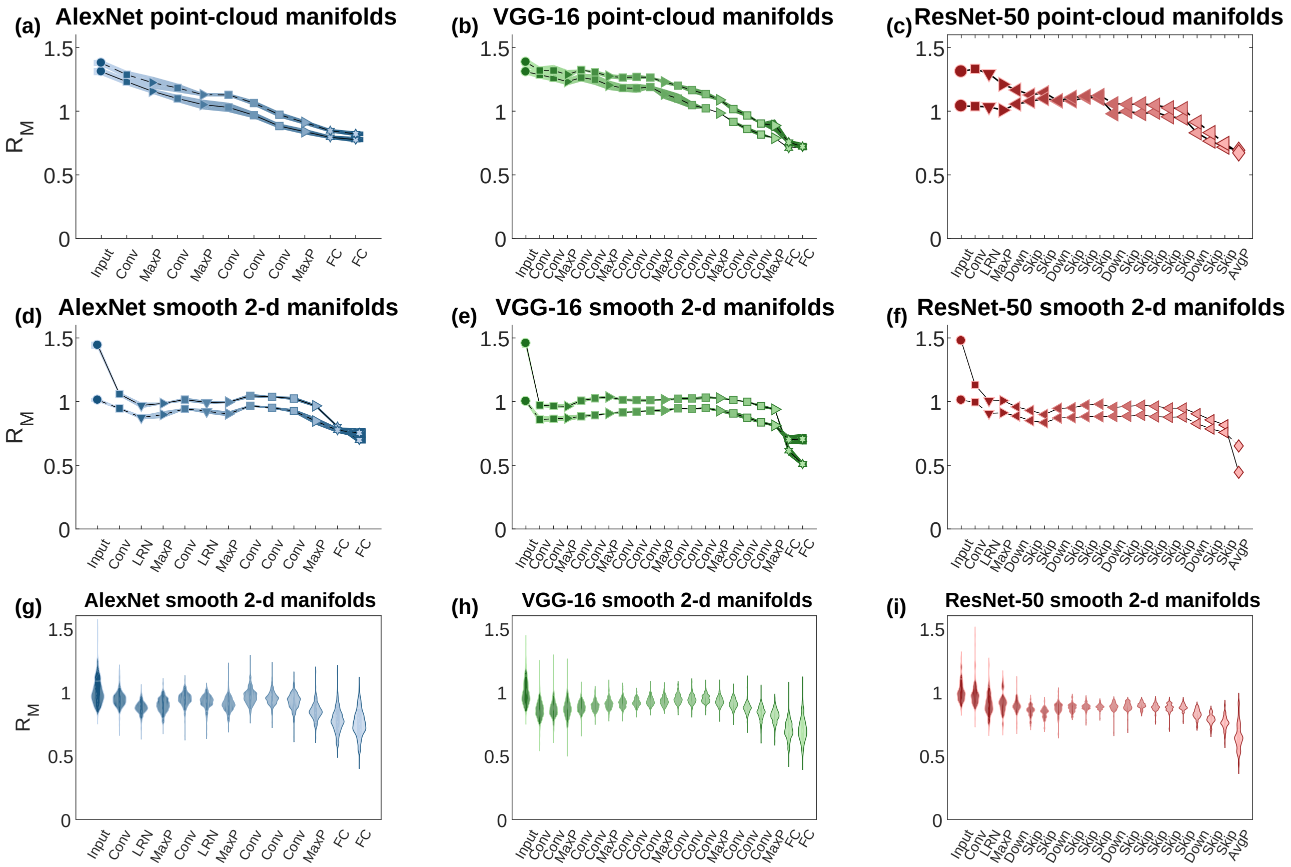

Figure 6: Manifold radii along the hierarchy.
(a-c) Mean manifold radius for point-cloud manifolds of AlexNet (a), VGG-16 (b) and ResNet-50 (c); full-line: full-class manifolds, dashed-line: top 10% manifolds. (d-f) Mean manifold radius for smooth 2-d manifolds of AlexNet (d), VGG-16 (e) and ResNet-50 (f); full-line: translation manifolds, dashed-line: shear manifolds. (g-i) Distribution of manifold radius for smooth 2-d shear manifolds for the same deep networks as (d-f), illustrated as per-layer histogram (kernel width is std/5).
Line and markers at (a,b,d,e) indicate mean value over different choices of objects and surrounding shaded areas at indicate 95% confidence interval, while at (c,f) only the first set of objects used. The x-axis labels provides abbreviation of the layer types. Marker shape represents layer type (circle- pixel layer, square- convolution layer, right-triangle- max-pooling layer, hexagon- fully connected layer, diamond- average pooling layer, down-triangle- local normalization layer, left-triangle- a skip module). Features in linear layers are extracted after a ReLU non-linearity. Color (blue- AlexNet, green- VGG-16, red- ResNet-50) changes from dark to light along the network.

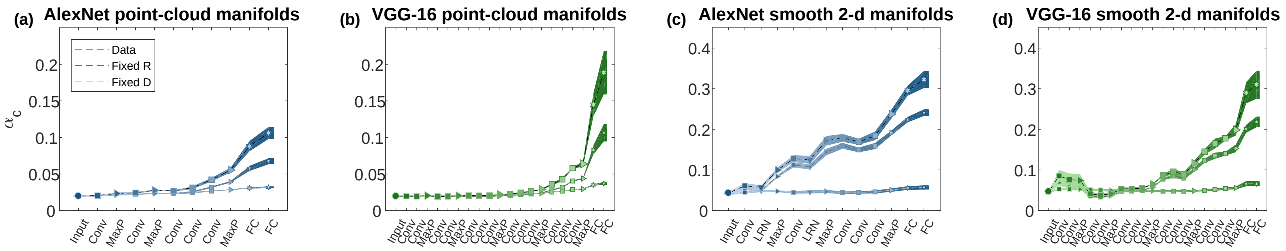

Figure 7: Capacity changes relation to radii and dimensions changes. Classification capacity for point-cloud manifolds (top-10% class) along the layers of AlexNet (a), VGG-16 (b) and for smooth 2-d shear manifolds along the layers of AlexNet (c), VGG-16 (d). The results using the full data (block lines), previously presented in main figures 4 and 5, are to be compared to the expected capacity with the observed dimensions but the radii fixed to their value at the pixel layer (dark gray lines), or the expected capacity with the observed radii but the dimensions fixed to its value at the pixel layer (light gray lines). Shared areas represent 95% confidence intervals with respect to sampling of different object.
The x-axis labels provides abbreviation of the layer types. Marker shape represents layer type (circle- pixel layer, square- convolution layer, right-triangle- max-pooling layer, hexagon- fully connected layer, diamond- average pooling layer, down-triangle- local normalization layer, left-triangle- a skip module). Features in linear layers are extracted after a ReLU non-linearity. Color (blue- AlexNet, green- VGG-16) changes from dark to light along the network.

1.1.4 Geometry of 1-d vs 2-d smooth manifolds in AlexNet, VGG-16, ResNet-50

As a measure for how complicated the internal representations of each manifold is, we measure a fraction between manifold dimensions created by 2-d variation and 1-d variation in the latent parameter space (figure 8a-c). When D_M^{2d}/D_M^{1d} is 2, it means that the dimensionality (in neural space) of 2-d variation is exactly twice the dimensionality created by 1-d variation. This can be either because the subspaces created by 2-d variation are factorized, or because the created manifold is small. If D_M^{2d}/D_M^{1d} is large (above 2), that means that there is a nonlinear interaction between the dimensions created by the 2-d variations in the latent space, and additional dimensions are created as a result. Here we find this is the case when manifold radii is large enough, as evident for translation manifolds which are very high-dimensional at the pixel layer. On the other hand, R_M^{2d}/R_M^{1d} is 1 throughout this data-set, as expected when the variability induced by each of 1-d transformation is arranged in orthogonal axes (figure 8d-f).

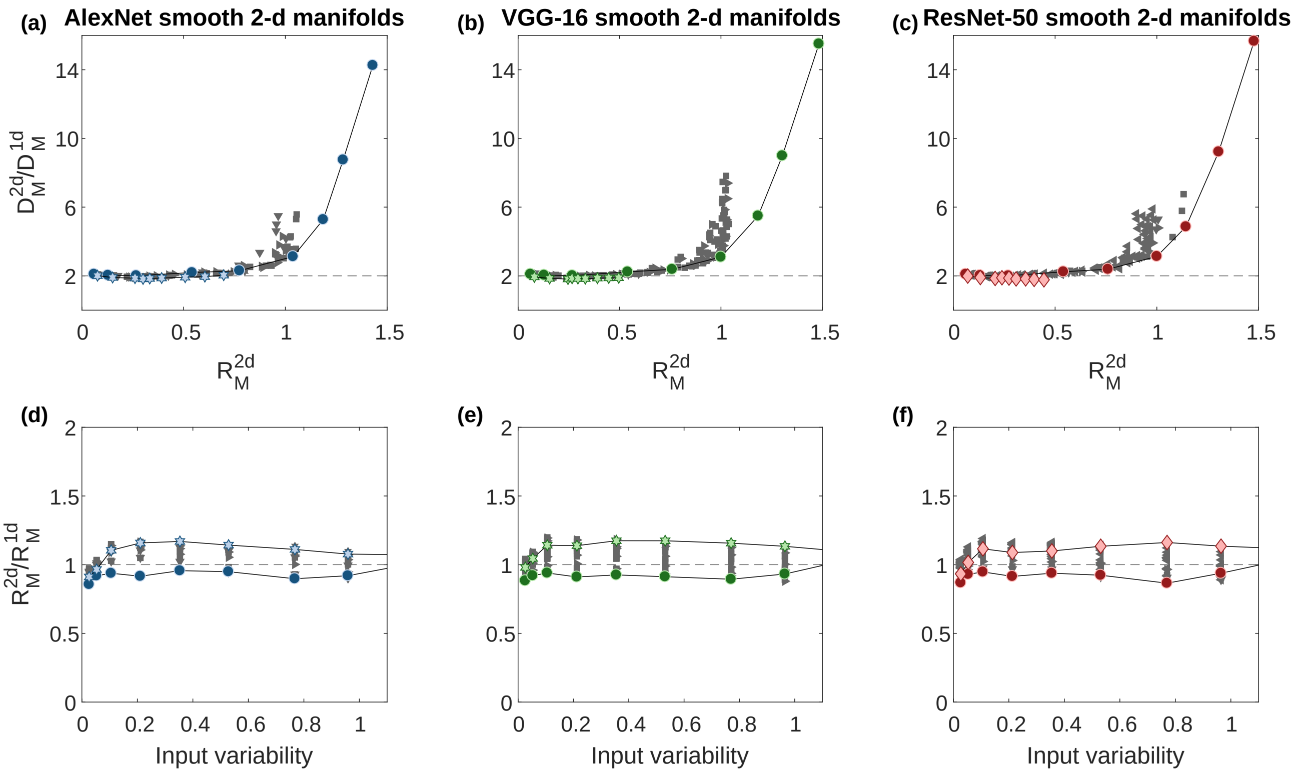

Figure 8: Comparison of geometry of 2-d and 1-d manifolds.
(a-c) Manifolds dimensions for smooth 2-d manifolds using AlexNet (a), VGG-16 (b) and ResNet-50 (c) relative to the average of the dimensions of corresponding 1-d manifolds with the same maximal object displacement (y-axis), shown vs the 2-d manifolds radii (x-axis).
(d-f) Manifolds radii for smooth 2-d manifolds using AlexNet (d), VGG-16 (e) and ResNet-50 (f) relative to the average of the radii of corresponding 1-d manifolds (y-axis), shown vs the 2-d stimuli variability, measured using SI equation (3) at the pixel layer (x-axis).
Marker shape represents layer type (circle- pixel layer, square- convolution layer, right-triangle- max-pooling layer, hexagon- fully connected layer, diamond- average pooling layer, down-triangle- local normalization layer, left-triangle- a skip module). Colored results are from first and last layers, all others layers are in gray to avoid clutter.

1.1.5 Manifold correlations and their effect on capacity in AlexNet, VGG-16, ResNet-50

Here we demonstrate that different types of between-manifold correlations (i.e. center-center, and axis-axis) are reduce across the layers of the deep hierarchies analyzed, on both point-cloud and smooth manifolds (figure 9). Consider P manifolds which correspond to the response of N neurons to M samples, denoted $F^{\mu}_{i,m}$ for $\mu = 1..P$, $i = 1..N$ and $m = 1..M$. For each μ a manifold is described by its center $\vec{x}^{\mu}$ and singular value decomposition (SVD) $F^{\mu}_{i,m} = x^{\mu}_i + \Sigma_l \lambda^{\mu}_l u^{\mu,l}_i v^{\mu,l}_m$ for non-negative scalars $\{\lambda^{\mu}_l\}_l$ and orthonormal set of vectors $\{\vec{u}^{\mu,l}\}_l$.
Then center correlations are defined

$$\rho_{CC} = \left\langle \frac{|\vec{x}^{\mu} \cdot \vec{x}^{\nu}|}{||\vec{x}^{\mu}|| \cdot ||\vec{x}^{\nu}||} \right\rangle_{\mu \neq \nu} \tag{1}$$

and axes correlations are defined

$$\rho_{AA} = \left\langle \sum_l \hat{\lambda}^{\mu}_l \hat{\lambda}^{\nu}_l |\vec{u}^{\mu,l} \cdot \vec{u}^{\nu,l}| \right\rangle_{\mu \neq \nu} \tag{2}$$

where $\hat{\lambda}^{\mu}_l = \lambda^{\mu}_l / \sqrt{\sum_l (\lambda^{\mu}_l)^2}$. Furthermore, the total manifold variability is defined:

$$v = \left\langle \frac{\sqrt{\sum_l (\lambda^{\mu}_l)^2}}{||\vec{x}^{\mu}||} \right\rangle_{\mu} \tag{3}$$

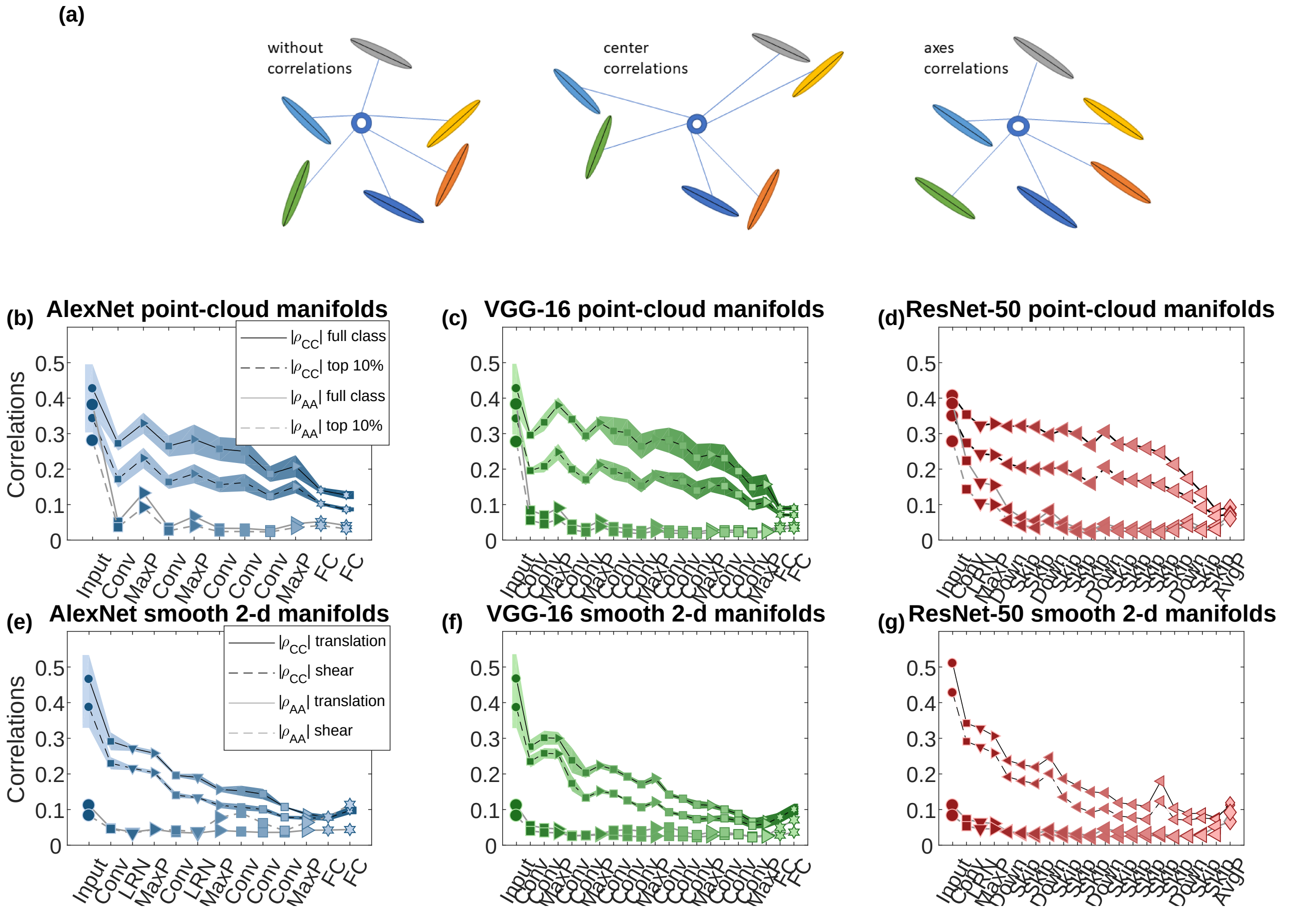

Figure 9: Center and axes correlations between manifolds. (a) illustration of object manifolds without correlations (left), where centers are randomly sampled points and the main axis of variation are randomly oriented; with center correlations (middle), where centers are clustered; with axes correlations (right), where the main axis of variation are aligned between different manifolds.
(b-d) Center and axes correlations for point-cloud manifolds using AlexNet (b), VGG-16 (c) and ResNet-50 (d); black- center correlations; gray- axes correlations; full line- full class manifolds, dashed line- top 10% manifolds.
(e-g) Center and axes correlations for smooth manifolds using AlexNet (e), VGG-16 (f) and ResNet-50 (g); black- center correlations; gray- axes correlations; full line- translation manifolds, dashed line- shear manifolds.
For center correlations of AlexNet and VGG-16 line and markers indicate mean value over different choices of objects; surrounding shaded areas indicate 95% confidence interval, with some of the results previously presented as "after training" results of main figure 7. For ResNet-50 only the first set of objects were used. The x-axis labels provides abbreviation of the layer types. Marker shape represents layer type (circle- pixel layer, square- convolution layer, right-triangle- max-pooling layer, hexagon- fully connected layer, diamond- average pooling layer, down-triangle- local normalization layer, left-triangle- a skip module). Features in linear layers and skip modules are extracted after a ReLU non-linearity. Color (blue- AlexNet, green- VGG-16, red- ResNet-50) changes from dark to light along the network.

Furthermore, comparing capacity from the data manifolds with capacity on surrogate data where different types of correlations are removed through randomization allows us to quantify that center-center correlations have a substantial effect while axes-axes correlations have a much smaller effect (figure 10). As predicted by theory, center-correlations decrease capacity while axes correlations increase capacity, relative to a correlations-free baseline.

Creation of data without center correlations is done by replacing the center of each manifold with a random vector. Creation of data without axes correlations is done by applying a random permutation to the neurons of each manifold. Then by comparing numerically calculated capacity (Methods in the main text) from the original data to that calculated on the surrogate data the effect of the removed correlations is revealed.

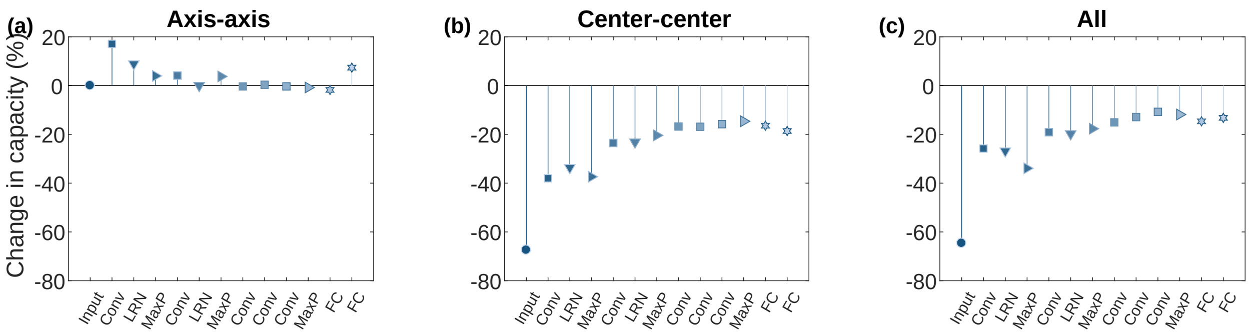

Figure 10: Quantifying correlations' effects on capacity through randomization. Change of numerically measured capacity due to a randomization procedure which produce manifolds where different kinds of correlations is absent. Results shown as percent changes of original data capacity relative to capacity of the randomized data. All results are calculated from smooth 1-d shear manifolds.
(a) The effect of removing axis-axis correlations at different layers of AlexNet.
(b) The effect of removing center-center correlations at different layers of AlexNet.
(c) The effect of removing both correlations at different layers of AlexNet.
The x-axis labels provides abbreviation of the layer types. Marker shape represents layer type (circle- pixel layer, square- convolution layer, right-triangle- max-pooling layer, hexagon- fully connected layer, diamond- average pooling layer, down-triangle- local normalization layer, left-triangle- a skip module). Features in linear layers and skip modules are extracted after a ReLU non-linearity. Color changes from dark to light along the network.

1.1.6 Deep network building-blocks have a consistent effect on manifold geometry and correlations

As discussed in the main text, the role of the different network building-blocks is explored by analyzing the effect on capacity of single operations and computational building-blocks, i.e. common operation sequences used in DCNNs (figure 8 in the main text). Figure 11 complement this discussion by exhibiting results for linear operations (i.e. convolutional and fully-connected layers) and partial building blocks (convolution followed by ReLU, without terminating max-pooling operation as in the main text). On both cases the change due to those building blocks is much less consistent than that of the full sequence.

On the other hand, a similar analysis of the effect of a "skip module", a sequence of operations used in ResNet-50, usually reduces the dimension, radius and correlations, with a clear trade-off effect (where cases where dimension is not reduced are associated with reduction of correlations and vice-versa). Indeed those skip modules replace in "Residual Networks" the use of convolutions followed by ReLU and max-pooling operations [1].

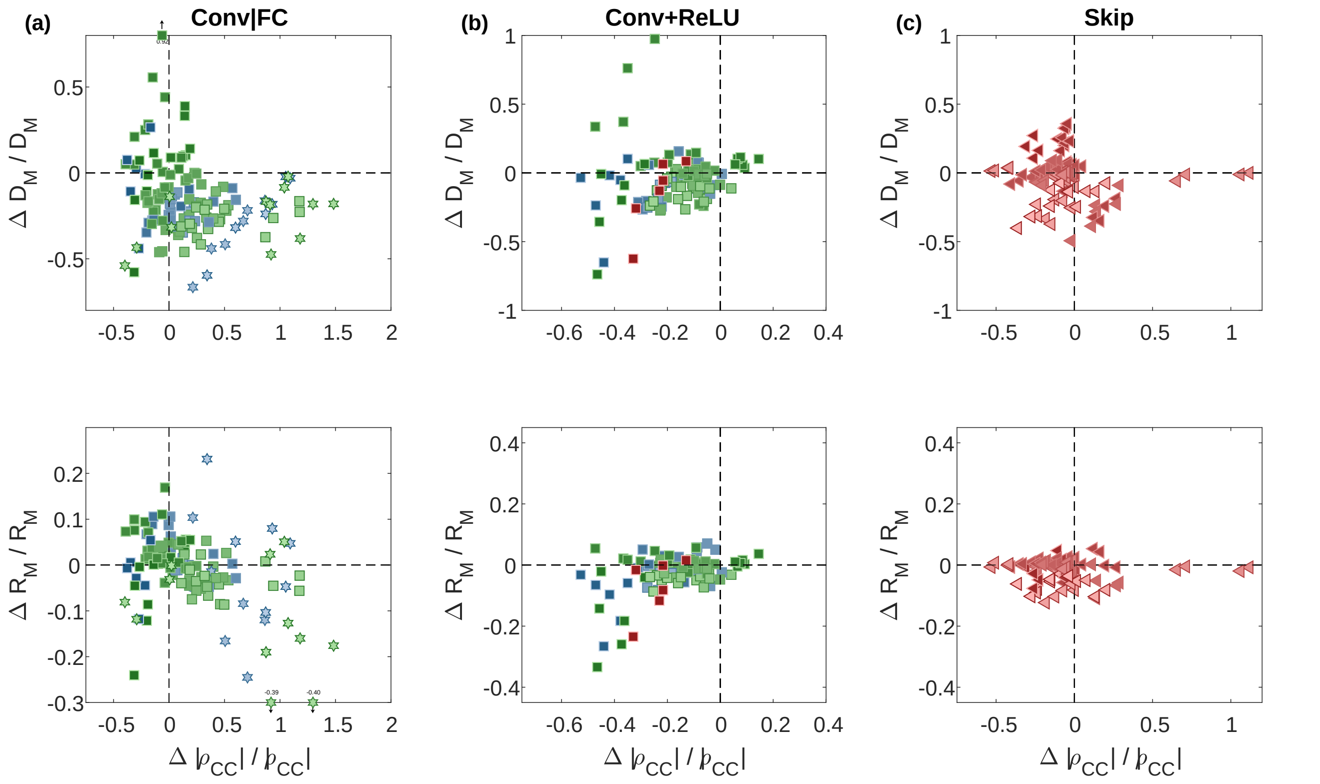

Figure 11: Manifold property changes by network building blocks. Changes in the relative manifold properties between the input and the output of different network building-blocks, shown as change in dimension vs change in center correlations (top) and change in radius vs change in center correlations (bottom). Each panel pools results from a specific building-block in AlexNet (blue markers), VGG-16 (green markers) and ResNet-50 (red markers) for both point-cloud manifolds (full class, top 10%) and smooth manifolds (1-d and 2-d, translation and shear). Marker shape represents layer type (square- convolution layer, hexagon- fully connected layer, left-triangle- a skip module). Color changes from dark to light along the network.
(a) Changes in manifold properties for isolated linear operations.
(b) Changes in manifold properties for a common sequence of layers, convolution followed by ReLU operation.
(c) Changes in manifold properties for "skip modules", a common sequence in the ResNet-50 architecture.

1.2 Predictions of manifold separability theory

In this section we demonstrate that the theory of linear separability of manifolds provide interesting predictions which may be verified in manifold created by the layers of DCNNs.

1.2.1 Comparison between theory and numerically measured capacity in smooth manifolds of AlexNet, VGG-16, ResNet-50

Here we demonstrate that the manifold capacity computed from the full mean field theory (main text equation (1)) matches capacity measured numerically (Methods in main text). Figure 12 presents an excellent match across all layers and manifold variability levels using different DCNNs.

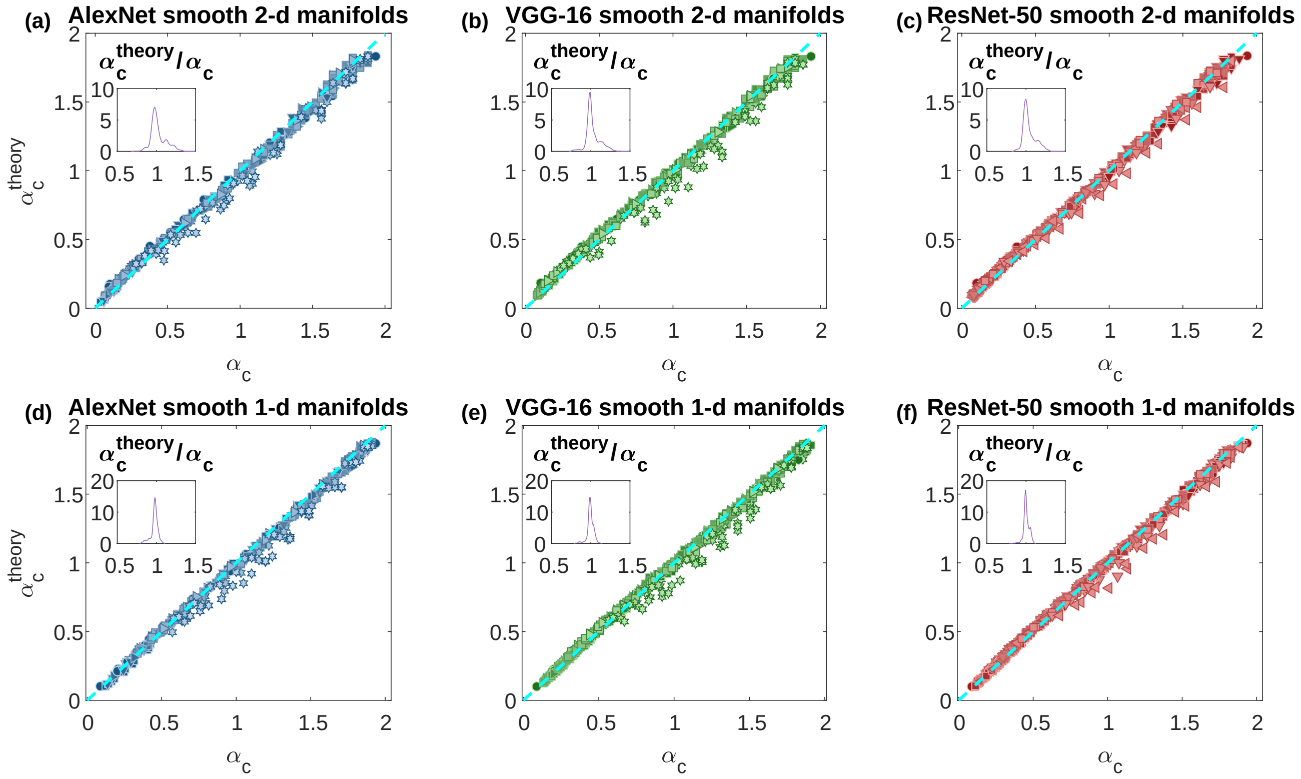

Figure 12: Comparison of capacity measured using theory and numerically for smooth manifolds. Comparison of numerically measured capacity (x-axis) with the theoretical prediction (y-axis) for smooth manifolds at different layers along the hierarchy and different manifold variability levels. Inset: histogram of the ratio between the y-axis and the x-axis.
(a-c) Results for smooth 2-d manifolds across AlexNet (a), VGG-16 (b) and ResNet-50 (c)
(d-f) Results for smooth 1-d manifolds across AlexNet (d), VGG-16 (e) and ResNet-50 (f)
Marker shape represents layer type (circle- pixel layer, square- convolution layer, right-triangle- max-pooling layer, hexagon- fully connected layer, diamond- average pooling layer, down-triangle- local normalization layer, left-triangle- a skip module). Color changes from dark to light along the network.

1.2.2 Manifold capacity's dependence on number of objects and neurons

As discussed in the main text, while the theory is derived for large number of objects P and number of neurons N, meaningful results are achieved already at reasonable values.

Main figure 9c-d exhibits that measuring capacity using a finite number of objects shows only a small dependence on the number of objects used, and that already for $P \approx 50$ the measured capacity is a good approximation for the asymptotic value. Figure 13 shows similar results for 1-d and 2-d smooth manifolds, and using different levels of manifold variability.

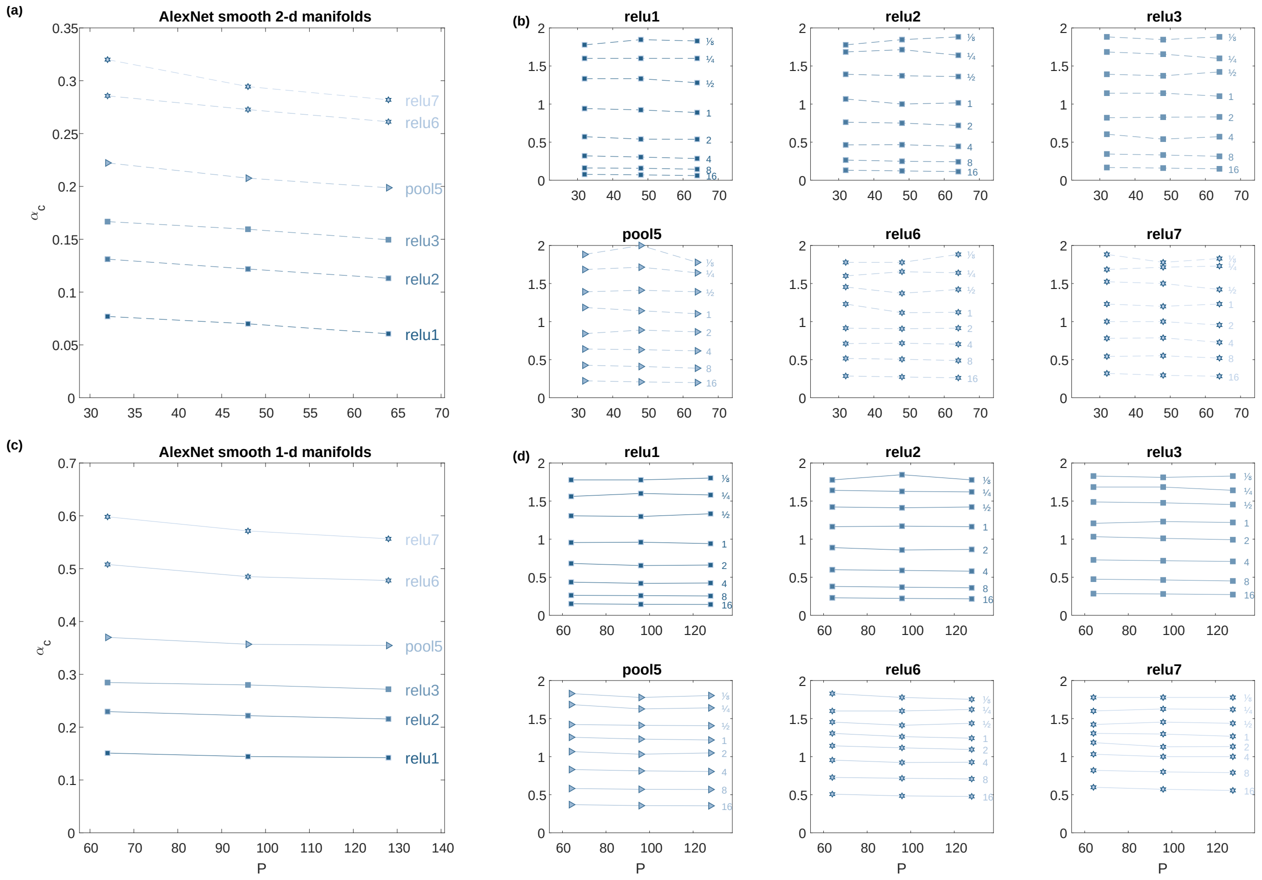

Figure 13: Extensive capacity in smooth manifolds. Numerically measured capacity (y-axis) at different number of objects (x-axis) for smooth manifolds at different layers and different levels of manifold variability.
(a-b) Results for 2-d shear manifolds at 6 layers across AlexNet and 8 levels of manifold variability. (a) all 6 layers at the largest variability level. (b) each of the 6 layers at 8 different levels of variability, indicated (text) as the maximal displacement of the object corners.
(c-d) Results for 1-d translation manifolds at 6 layers across AlexNet and 8 levels of manifold variability. (c) all 6 layers at the largest variability level. (d) each of the 6 layers at 8 different levels of variability, indicated (text) as the maximal displacement of the object corners.
Marker shape represents layer type (circle- pixel layer, square- convolution layer, right-triangle- max-pooling layer, hexagon- fully connected layer, diamond- average pooling layer, down-triangle- local normalization layer, left-triangle- a skip module). Color changes from dark to light along the network.

Similarly, when using mean-field algorithms to measure capacity and geometric properties using a finite number of neurons we expect the result to have only a small dependence on the number of neurons used. Figure 14 shows this is the case, as already when using few hundred neurons a good approximation of the asymptotic value is achieved.

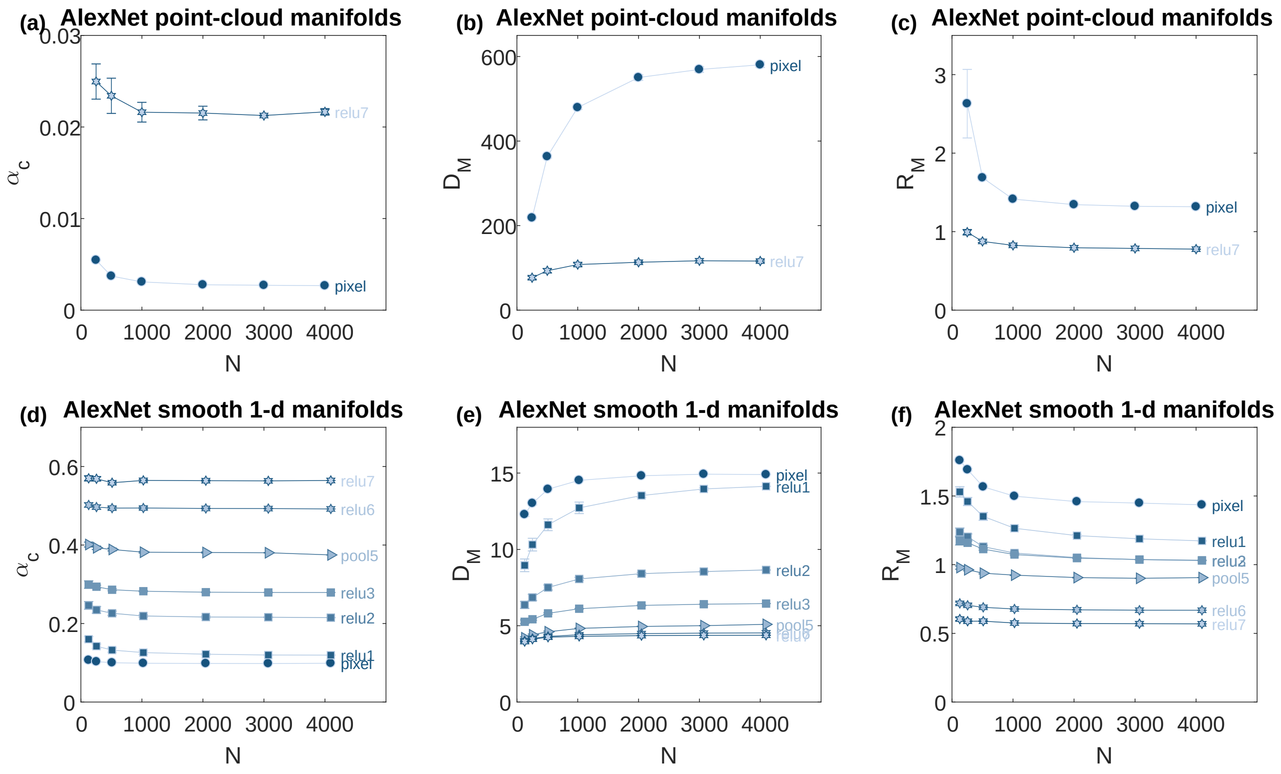

Figure 14: Extensivity of capacity and manifold geometric properties. Measuring manifold capacity and properties by subsampling different number of neurons; values are averaged across subsampling sets (5 for point-cloud manifolds, 10 for smooth manifolds); error-bars indicate standard deviation.
(a-c) Results for top 10% point-cloud manifolds (y-axis) vs the number of subsampled neurons (x-axis).
(a) classification capacity; (b) manifold dimension; (c) manifold radius
(d-f) Results for smooth 1-d translation manifolds (y-axis) vs the number of subsampled neurons (x-axis).
(d) classification capacity; (e) manifold dimension; (f) manifold radius
Marker shape represents layer type (circle- pixel layer, square- convolution layer, right-triangle- max-pooling layer, hexagon- fully connected layer, diamond- average pooling layer, down-triangle- local normalization layer, left-triangle- a skip module). Color changes from dark to light along the network.

1.2.3 Comparison between full theory and approximation with geometric properties for capacity of smooth manifolds of AlexNet, VGG-16, ResNet-50

Here we demonstrate that the manifold capacity computed from the full mean field theory (main text equation (1)) is well approximated by the estimated manifold capacity using the expression provided in Methods, main text equations (3)-(5), namely, the capacity of L_2 balls, whose radius and dimension are equivalent to the manifold's effective radius and dimension computed the anchor points, i.e. $\alpha_c \approx \alpha_{Balls}(R_M, D_M)$ (Methods, [2]). The expression for the manifold capacity of L_2 balls is givne by:

$$\alpha_{\text{Ball}}^{-1}(R, D) = \int_{-\frac{\sqrt{D}}{R}}^{R\sqrt{D}} Dt_0 \frac{(R\sqrt{D} - t_0)^2}{R^2 + 1} + \int_{-\infty}^{-\frac{\sqrt{D}}{R}} Dt_0 (t_0^2 + D) \tag{4}$$

with Gaussian measure $Dt_0 = \frac{1}{\sqrt{2\pi}} e^{-\frac{t_0^2}{2}}$, which was first introduced in [3]. The agreement between α_c and $\alpha_{Balls}(R_M, D_M)$ is shown in various networks for smooth manifolds (figure 15).

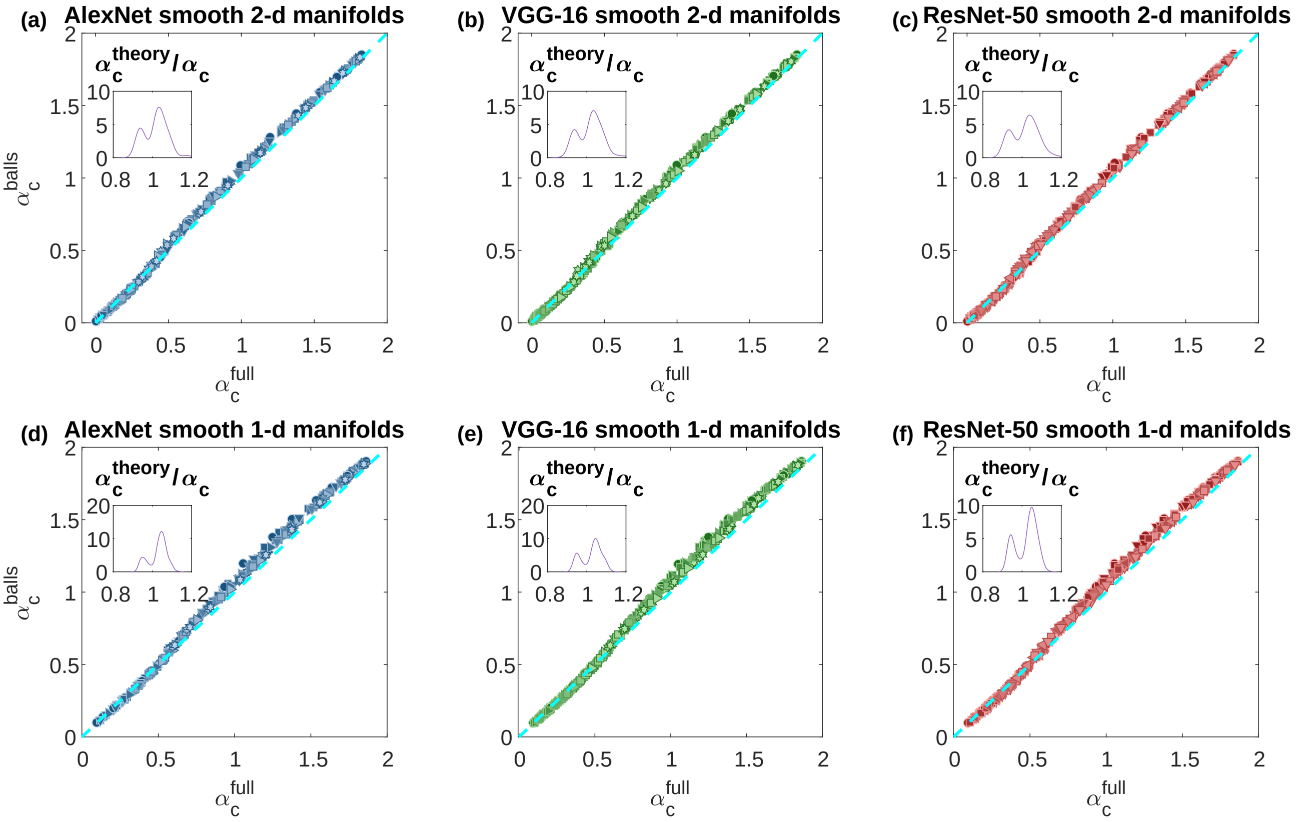

Figure 15: Comparison of capacity measured using full theory and balls approximation for smooth manifolds.
Comparison o capacity measured using the full theory (x-axis) with the approximation based on balls capacity and geometric properties (y-axis) for smooth manifolds at different layers along the hierarchy and different manifold variability levels. Inset: histogram of the ratio between the y-axis and the x-axis.
(a-c) Results for smooth 2-d manifolds across AlexNet (a), VGG-16 (b) and ResNet-50 (c)
(d-f) Results for smooth 1-d manifolds across AlexNet (d), VGG-16 (e) and ResNet-50 (f)
Marker shape represents layer type (circle- pixel layer, square- convolution layer, right-triangle- max-pooling layer, hexagon- fully connected layer, diamond- average pooling layer, down-triangle- local normalization layer, left-triangle- a skip module). Color changes from dark to light along the network.

1.2.4 Random sampling versus random projection

In order to numerically measure capacity, the ability to linearly separate object manifolds is tested using different number of neurons (Methods in main text). To do so we first subsample the features from an original high-dimensional representations. Here we compare two popular methods for subsampling features: random subsampling and random projection. Using smooth manifolds, we compare the numerical capacity measured by random subsampling and random projection. Comparisons of the measured capacity values using either sampling method types are shown in figure 16a and b for 1-d and 2-d smooth manifold, respectively. The good agreement between the two methods is demonstrated by the alignment of most points to the identity diagonal (dashed cyan line). A notable deviation from this agreement is evident in the above-diagonal square markers where capacity measured through subsampling is above that measured through random projections; those correspond to the 'relu5' layer where features are very sparse. Figure 16c presents sparsity histogram for the neurons of each layer, showing how 'relu5' is the only layer where the mode of this histogram is near 1.

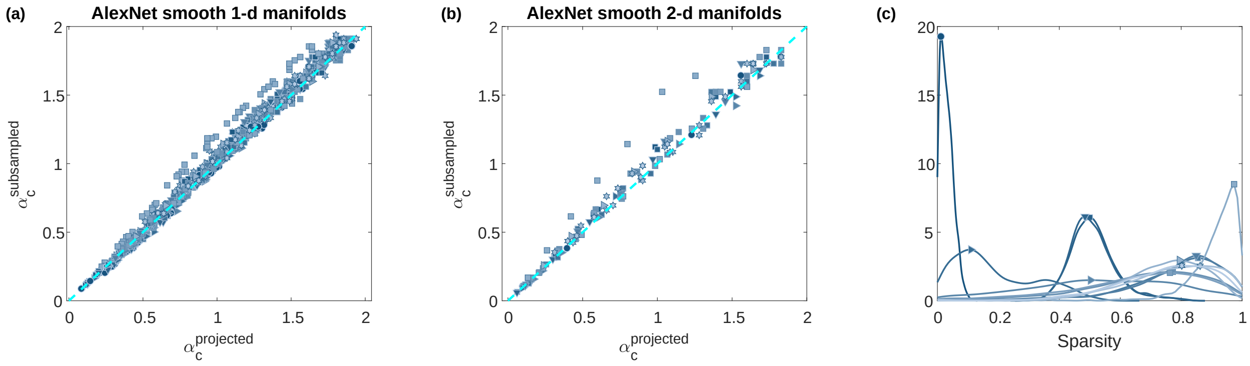

Figure 16: Capacity using subsampled vs projected features.
(a-b) Comparison of numerically measured capacity using random projections (x-axis) and using subsampling (y-axis) for AlexNet at different layers along the hierarchy and different levels of manifold variability.
(a) smooth 1-d manifolds (b) smooth 2-d manifolds
(c) histogram of per-neuron sparsity values for each layer; sparsity of a neuron is defined as the fraction of stimuli which elicit a zero response.
Marker shape represents layer type (circle- pixel layer, square- convolution layer, right-triangle- max-pooling layer, hexagon- fully connected layer, diamond- average pooling layer, down-triangle- local normalization layer, left-triangle- a skip module). Color changes from dark to light along the network.

2 Methods

2.1 Measuring capacity and geometric manifold properties

Here we develop a procedure to recover the common center correlations structure of general manifolds. Consider P manifolds in $\mathbb{R}^N$ and denote $X \in \mathbb{R}^{N \times P}$ a matrix with the their center of mass, we seek to decompose a center correlations matrix $C = X^T X \in \mathbb{R}^{P \times P}$ into a sum of diagonal part D and a low-rank part C_K per main text equation (6). To achieve this, given X we seek an orthonormal set $V \in \mathbb{R}^{N \times K}$ (i.e. $V^T V = I_K$) such that X in the null-space of V have approximately diagonal correlations:

$$\hat{X} = X - V\left(V^T X\right) \tag{5}$$

$$\hat{D} = \hat{X}^T \hat{X} \tag{6}$$

For specific K, this can be done by directly optimizing for V which both satisfies the orthonormality constraint $V^T V = I_K$ and minimize the following cost function (square_corrcoeff_cost() in Algorithm 1):

$$\text{cost}\left(X, V\right) = \frac{1}{2} \sum_{\mu \neq \nu} \hat{D}_{\mu\nu}^2 / \hat{D}_{\mu\mu} \hat{D}_{\nu\nu} \tag{7}$$

where we note that $\hat{D}$ which minimize this cost is expected to be approximately diagonal. This non-convex optimization problem can be efficiently solved using the algorithm and code from [4].

When K is not known in advance, it can be found iteratively by taking increasing values for K, until the cost no longer decrease. To improve the stability of the algorithm we are using V_K as initial value for the optimization when optimizing for V_{K+1}; specifically we take an initial value of $[V_K|v]$ where v is randomly sampled vector and repeat the procedure several times to overcome dependencies on the initial conditions in this non-convex optimization. This approach achieves a minimal value for the cost at intermediate value of K, so that it has no free parameter. Furthermore, the complexity of this procedure does not depend on the dimension N as when $N \geq P$ the centers are of rank $P-1$ (as without loss of generality the global mean is 0) and thus can be expressed in $P-1$ coordinates. The full procedure is finding the residual data in the null-space of the common components is described in Algorithm 1. Once the common components are removed, classification capacity, as well as manifold geometrical properties (R_M and D_M, see main text) can be estimated from the residual manifolds using the methods described in [3]; the full procedure is described in Algorithm 2.

Algorithm 1 find_common_components: find the number and directions of common components in correlated data

Function V^*, X_K^* =find_common_components(X)
Input: data $X \in \mathbb{R}^{N\times P}$
Output: common components matrix $V^* \in \mathbb{R}^{K\times N}$, data in the null-space of the common components $X_K^* \in \mathbb{R}^{N\times P}$
Parameters: maximal K to try (a number smaller than P) and at the minima of which target function to stop (a boolean choosing mean square correlations or the mean absolute correlations)

1. Find an orthonormal set $Q \in \mathbb{R}^{N\times(P-1)}$ which span the data, represent it in coordinates of Q as $X_Q = Q^T X \in \mathbb{R}^{(P-1)\times P}$
2. Initialize $V = [\cdot]$ and the target function to ∞
3. Initialize $K = 0$, and until the maximal K is reached or the target hasn't improved for 3 iterations:
   (a) Initialize $z = 0$
   (b) Repeat the optimization 10 times with different initial conditions to find the result with the smallest change to the norms:
      i. Sample $\{s_\mu \sim \mathcal{N}(0,1)\}_{\mu=1}^{P}$ and set initial value $V_0 = [V|X_Q\vec{s}]$
      ii. Find V_1 which minimize the square cost function under the constraint $V_1^T V_1 = I$ with initial value V_0: $V_1 = \mathrm{OptStiefelGBB}\left(V_0, \mathrm{square\_corrcoeff\_cost}\right)$
      iii. Calculate residual data $\vec{x}_K^\mu = \vec{x}^\mu - V\left(V^T\vec{x}^\mu\right)$
      iv. Calculate $z_1 = \min_\mu \|\vec{x}^\mu\| / \|\vec{x}_K^\mu\|$
      v. If $z_1 > z$ save the results $V = V_1$
   (c) Calculate residual centers $X_K = X_Q - V\left(V^T X_Q\right)$
   (d) Calculate the target function (mean square correlations or mean absolute correlations)
   (e) If the current target function is lower than the best results so far, update the best results $K^* = K$,$V^* = V$, $X_K^* = X_K$
4. Translate the results to original coordinates $V^* = QV^*$, $X_K^* = QX_K^*$

Function cost=square_corrcoeff_cost(V,X)
Input: orthogonal matrix $V \in \mathbb{R}^{N\times K}$ and data matrix $X \in \mathbb{R}^{N\times P}$
Output: sum of the squares of the off-diagonal normalized correlations between the vectors of X in the null-space of V

1. $C = X^T X$
2. $\hat{X} = X^T V$
3. $\hat{D}_{\mu\nu} = C_{\mu\nu} - \sum_i \hat{X}_{\mu i}\hat{X}_{\nu i}$
4. cost $= \frac{1}{2}\sum_{\mu\nu} \hat{D}_{\mu\nu}^2 / \hat{D}_{\mu\mu}\hat{D}_{\nu\nu}$

Function V=OptStiefelGBB(cost$(\cdot)$, V_0): curvilinear search algorithm for optimization on Stiefel manifold
Input: a cost function cost(V) and initial value $V_0 \in \mathbb{R}^{N\times K}$
Output: $V \in \mathbb{R}^{N\times K}$ which minimize cost(V) under the constraint $V^T V = I$
See [4]

Algorithm 2 correlated_manifolds_geometry: Geometric properties for correlated manifolds

Function correlated_manifolds_geometry($\{F^\mu\}$)
Input: Neural object responses $\left\{F_i^\mu \in \mathbb{R}^N\right\}_{i\in[1..M_\mu]}^{\mu=1..P}$

1. Project data to the null-space of the common components: $\{f^\mu\} = \text{find\_residual\_data}\left(\{F^\mu\}\right)$
2. For $\mu = 1..P$, calculate geometry $D_M^\mu, R_M^\mu, \alpha_c^\mu = \text{manifold\_geometry}\left(f^\mu\right)$

Output: $\{D_M^\mu\}_{\mu=1}^P$, $\{R_M^\mu\}_{\mu=1}^P$, $\{\alpha_c^\mu\}_{\mu=1}^P$
Function $\{f^\mu\}$=find_residual_data($\{F^\mu\}$)
Input: neural response function with N neurons, P objects, M_μ samples per object $\left\{F_i^\mu \in \mathbb{R}^N\right\}_{i=1..M_\mu}^{\mu=1..P}$
Output: residual neural response function $\left\{f_i^\mu \in \mathbb{R}^N\right\}_{i=1..M_\mu}^{\mu=1..P}$

1. Remove the global mean from the input F
2. Calculate object centers $X \in \mathbb{R}^{N\times P}$
3. Find the common components $V = \text{find\_common\_components}\left(\text{X}\right)$
4. Use V to find the neural response in the null-space of V: $f^\mu = F^\mu - V\left(V^T F^\mu\right)$

Function manifold_geometry(f^μ)
Input: Manifold $\left\{f_i^\mu \in \mathbb{R}^N\right\}_{i=1..M_\mu}$
Parameters: Numbers of Gaussian samples N_G
Output: manifold geometry D_M^μ,R_M^μ,α_c^μ
See [2] supplementary material, algorithms 2, 4.

2.2 Measuring manifold capacity numerically

Algorithm 3 numerical_capaity: Measure numerical capacity

Function: numerical_capaity($\{F_i^\mu\}$)
Input: neural response function with N neurons, P objects, M_μ samples per object $\left\{F_i^\mu \in \mathbb{R}^N\right\}_{i=1..M_\mu}^{\mu=1..P}$
Output: numerical capacity α_c

1. Binary search for n where fraction_separable_dichotomies$\left(\{F_i^\mu\}, n\right)$ surpass 0.5
2. Report $\alpha_c = P/n$

Function fraction_separable_dichotomies($\{F_i^\mu\}$, n): measure the fraction of separable random dichotomies
Input: neural response function with N neurons, P objects, M_μ samples per object $\left\{F_i^\mu \in \mathbb{R}^N\right\}_{i=1..M_\mu}^{\mu=1..P}$ and an integer $n \in [0..N]$
Output: the fraction of linearly separable dichotomies after projecting the input to n
Parameters: $N_{dichotomies}$ the number of dichotomies to sample

1. Repeat $N_{dichotomies}$ times:
   (a) Sample random projection matrix $B \in \mathbb{R}^{n\times N}$ and set $f^\mu = BF^\mu\ \forall\mu = 1..P$
   (b) Sample random labeling $y \in \{\pm 1\}^P$
   (c) Use quadratic optimization or the method from [5] to check if there exists $w \in \mathbb{R}^n$ such that $y^\mu w^T f_i^\mu \geq 0\ \forall\mu = 1..P\ \forall i = 1..M_\mu$
2. Report the fraction of separable dichotomies

2.3 ImageNet classes used for point-cloud manifolds

The following ImageNet classes (a total of 50) were used as the first set of objects in the analysis of point-cloud manifolds.

moving van (n03796401)
hornbill (n01829413)
Tibetan terrier (n02097474)
microphone, mike (n03759954)
terrapin (n01667778)
wheelbarrow (n02797295)
race car (n04037443)
Weimaraner (n02092339)
knot (n03627232)
folding chair (n03376595)
sunscreen (n04357314)
coffeepot (n03063689)
overskirt (n03866082)
fox squirrel (n02356798)
standard poodle (n02113799)
oscilloscope (n03857828)
head cabbage (n07714571)
jeweler's loupe (n03692522)
bottlecap (n02877765)
llama (n02437616)
ruddy turnstone (n02025239)
hermit crab (n01986214)
ibex (n02417914)
vase (n04522168)
little blue heron (n02009229)
soda bottle (n03983396)
rotisserie (n04111531)
microwave oven (n03761084)
breastplate (n02895154)
home theater (n03529860)
black widow (n01774384)
mailbox (n03710193)
crutch (n03141823)
bighorn sheep (n02415577)
hartebeest (n02422106)
padlock (n03874599)
sewing machine (n04179913)
mountain bike (n03792782)
giant panda (n02510455)
flowerpot (n03991062)
bow (n02879718)
triceratops (n01704323)
otter hound (n02091635)
chain (n02999410)
cowboy hat (n03124170)
tripod (n04485082)
bell (n03017168)
smoothing iron (n03584829)
teapot (n04398044)
birdhouse (n02843684)

3 Notes

3.1 Theory for low-rank center correlations

Uncorrelated general manifolds Consider P manifolds $\{M^\mu\}_{\mu=1}^P$ which reside in $\mathbb{R}^N$ but have an intrinsic dimension of $D+1$:

$$M^\mu = \left\{ \sum_{l=0}^{D} s_l \vec{u}^{\mu,l} \ : \ \vec{s} \in S^\mu \right\} \tag{8}$$

where $S^\mu \subseteq \mathbb{R}^{D+1}$ are the manifold coordinates and the unit vectors $\left\{\vec{u}^{\mu,l} \in \mathbb{R}^N\right\}_{\mu=1..P}^{l=0..D}$ are the axes on which the (non-linear) manifold is defined. By convention $\vec{u}^{\mu,0}$ is the manifold center and s_0 is its norm in this parametrization. The manifold coordinates are defined by a set of functions $F_\mu(x) : \mathbb{R}^{D+1} \to \mathbb{R}$ for $\mu = 1..P$:

$$S^\mu = \{s \ : \ F_\mu(s) < 0\} \tag{9}$$

Denote $\alpha = \frac{P}{N}$ the system's load and we are interested in the case $N, P \to \infty$ with a finite α and ask when there exists a solution, that is a unit vector $\vec{w} \in \mathbb{R}^N$, a bias term $b \in \mathbb{R}$ such that:

$$y^\mu \left(w^T x + b\right) \geq 0 \ \forall x \in M^\mu \ \forall \mu = 1..P \tag{10}$$

The critical capacity α_c is the load value below it a solution is likely to exist and above it is not. Formally we consider the volume of the solution space under constraints of normalization of w (which we take as $\|w\|^2 = 1$) and satisfaction of the solution inequalities:

$$V[\alpha] = \int db \prod_i^N \int dw_i \delta\left(w^T w - 1\right) \prod_\mu^P \prod_{x \in M^\mu} \Theta\left(y^\mu \left(w^T x + b\right)\right) \tag{11}$$

where Θ is the Heaviside step function.

By assuming $\{u_i^{\mu,l}\}_{i=1..N}^{\mu=1..P,l=0..D}$ are i.i.d standard Gaussian variables $\mathcal{N}(0,1)$, thus avoiding any correlations between manifolds axes of variation and between manifold centers, and considering binary labeling $y^\mu \in \{\pm 1\}$ with a equal probability, it can be shown that the critical capacity satisfies [2], for homogeneous manifolds:

$$\alpha_S^{-1} \ = \ \int D^{D+1}\vec{t} \min_{\{\vec{v} \in \mathbb{R}^{D+1} \, : \, \forall \vec{s} \in S \ \ \vec{s} \cdot \vec{v}^\mu \geq 0\}} \|\vec{v}^\mu - \vec{t}^\mu\|^2 \tag{12}$$

where $Dt = dte^{-t^2/2}/\sqrt{2\pi}$ and for non-homogeneous manifolds:

$$\alpha_c^{-1} \ = \ \frac{1}{P} \sum_\mu^P \alpha_{S^\mu}^{-1} \tag{13}$$

Manifolds with full-rank center correlations Consider the more general case where the manifold center (or displacement relative to the origin) is correlated across manifolds, that is $\{\vec{u}^{\mu,0} \in \mathbb{R}^N\}_{\mu=1}^P$ have correlations. Denote those correlations:

$$C_{\mu\nu} \ = \ \frac{1}{N} \left[\vec{u}^{\mu,0} \cdot \vec{u}^{\nu,0}\right]_u \tag{14}$$

Assuming the correlations are of full rank, there exist unit vectors $\{\vec{r}^l \in \mathbb{R}^P\}_{l=1}^P$, and scalars $\{c_l \geq 0\}_{l=1}^P$ such that:

$$C_{\mu\nu} \ = \ \sum_l^P c_l r_\mu^l r_\nu^l \tag{15}$$

and denoting a matrix $L_{\mu\nu} = \sqrt{c_\nu} \vec{r}_\mu^\nu$ we have that:

$$C \ = \ \sum_l^P c_l \vec{r}^l \vec{r}^{lT} = LL^T \tag{16}$$

Now using $\{\phi_{li} \sim \mathcal{N}(0,1)\}_{l=1..P}^{i=1..N}$ i.i.d Gaussian variables we define a statistical model:

$$u_i^{\mu,0} = \sum_l^P \sqrt{c_l} r_\mu^l \phi_{li} \tag{17}$$

which satisfies SI equation (14).

Following [6],[2] the critical capacity can is characterized from the volume of solutions using the replica method; assuming replica symmetry we get an expression:

$$\alpha_c^{-1} = \int D^{(D+1)\times P} t_{l=0..D}^{\mu=1..P} \left[\min_{\vec{v}\in\mathcal{V}} \frac{1}{P} \sum_\mu^P \|\vec{v}^\mu - \vec{t}^\mu\|^2 \right]_y \tag{18}$$

$$\mathcal{V} = \left\{ \vec{v} \in \mathbb{R}^{(D+1)\times P} \ : \ \forall\mu \, \forall\vec{s}^\mu \in S^\mu \sum_{l=1}^D s_l v_l^\mu + y^\mu \sum_\nu L_{\mu\nu} v_0^\nu \geq 0 \right\} \tag{19}$$

where the matrix L couples the integration between different manifolds and the expression still depends on the labeling $\vec{y}$; compare to SI equation (13) from of the uncorrelated case written using a similar notation:

$$\alpha_c^{-1} = \int D^{(D+1)\times P} t_l^\mu \min_{\vec{v}\in\mathcal{V}} \frac{1}{P} \sum_\mu^P \|\vec{v}^\mu - \vec{t}^\mu\|^2 \tag{20}$$

$$\mathcal{V} = \left\{ \vec{v} \in \mathbb{R}^{(D+1)\times P} \ : \ \forall\mu \, \forall\vec{s}^\mu \in S^\mu \ \vec{s}^\mu \cdot \vec{v}^\mu \geq 0 \right\} \tag{21}$$

Manifolds with low-rank off-diagonal correlations For correlations with low-rank off-diagonal structure we assume there is a diagonal Δ with $\Delta_{\mu\mu} = d_\mu$ and C_K with rank $K \ll P$ such that:

$$C = \Delta + C_K \tag{22}$$

Noting that $\Delta^{-0.5} C_K \Delta^{-0.5}$ is symmetric there exists an orthonormal $U_K \in \mathbb{R}^{P\times K}$ and a diagonal $E_K \in \mathbb{R}^{K\times K}$ such that:

$$U_K E_K U_K^T = \Delta^{-0.5} C_K \Delta^{-0.5} \tag{23}$$

Denote U a completion of U_K to orthonormal basis and denote $E \in \mathbb{R}^{P\times P}$ defined as $E = \begin{bmatrix} E_K & 0 \\ 0 & 0 \end{bmatrix}$, we have:

$$C = \Delta^{0.5} \left(I + U_K E_K U_K^T \right) \Delta^{0.5} = \Delta^{0.5} U \left(I + E \right) U^T \Delta^{0.5} \tag{24}$$

so that $C = LL^T$ for:

$$L = \Delta^{0.5} U \left(I + E \right)^{0.5} \tag{25}$$

Using this notation capacity from SI equation (20) becomes:

$$\alpha_c^{-1} = \int D^{(D+1)\times P} t_l^\mu \left[\min_{\vec{v}\in\mathcal{V}} \left[\frac{1}{P} \sum_{l=0}^D \|\vec{v}_l - \vec{t}_l\|^2 \right] \right]_y \tag{26}$$

$$\mathcal{V} = \left\{ \vec{v} \in \mathbb{R}^{(D+1)\times P} \ : \ \forall\mu \, \forall\vec{s}^\mu \in S^\mu \sum_{l=1}^D s_l^\mu v_l^\mu + y^\mu \sqrt{d_\mu} \sum_\nu U_{\mu\nu} \sqrt{1+e_\nu} v_0^\nu \geq 0 \right\} \tag{27}$$

Now denote α_{ns} an approximation assuming the minima with respect to $\vec{v}_0$ is achieved for $v_0^\nu = 0$ for $\nu = 1..K$, and further neglect the contribution of the corresponding t_0^ν; as long as $K \ll P$ we can expect this approximation to be reasonable, yielding:

$$\alpha_{ns}^{-1} = \int D^{(D+1)\times P} t_l^\mu \left[\min_{\vec{v}\in\mathcal{V}} \left[\frac{1}{P} \sum_{l=1}^D \|\vec{v}_l - \vec{t}_l\|^2 + \frac{1}{P} \|\vec{v}_0 - t_0\|^2 \right] \right]_y \tag{28}$$

$$\mathcal{V} = \left\{ \vec{v} \in \mathbb{R}^{(D+1)\times P} \ : \ \forall\mu \, \forall\vec{s}^\mu \in S^\mu \sum_{l=1}^D s_l^\mu v_l^\mu + \sqrt{d_\mu} y^\mu \sum_{\nu=K+1}^P U_{\mu\nu} v_0^\nu \geq 0 \right\} \tag{29}$$

such that by change of variables $\vec{v}_0 \leftarrow \vec{y} \circ U \vec{v}_0$ and $\vec{t}_0 \leftarrow \vec{y} \circ U \vec{t}_0$ which does not affect the norm $\|\vec{v}_0 - t_0\|^2$ we have a decoupled expression:

$$\alpha_{ns}^{-1} = \int D^{(D+1)\times P} t_l^{\mu} \min_{\vec{v}\in\mathcal{V}} \left[\frac{1}{P}\sum_{l=0}^{D} \|\vec{v}_l - \vec{t}_l\|^2\right] \tag{30}$$

$$\mathcal{V} = \left\{\vec{v} \in \mathbb{R}^{(D+1)\times P} \,:\, \forall\mu\, \forall\vec{s}^{\mu} \in S^{\mu} \min_{\vec{s}\in S^{\mu}} \sum_{l=1}^{D} s_l v_l^{\mu} + \sqrt{d_{\mu}} v_0^{\mu} \geq 0\right\} \tag{31}$$

Thus by projecting the manifolds into the null-space of the directions associated with the non-diagonal part of the correlations matrix, we are back at the situation of manifolds with uncorrelated centers and thus can use capacity from SI equation (13) with appropriate scaling of the manifolds by the norm $\{d_{\mu}\}$.

3.2 Theory for manifolds of random point

Properties of point-cloud manifolds Consider a point-cloud manifold composed of M points $F = \left\{\vec{x}^m \in \mathbb{R}^N\right\}_{m=1}^{M}$ where N denote the ambiant dimension and assume the points reside in a $D+1$ affine subspace, such that there is an orthonormal set $\left\{u_l \in \mathbb{R}^N\right\}_{l=0}^{D}$ such that:

$$\vec{x}^m = s_0 \vec{u}_0 + \sum_{l=1}^{D} s_l^m \vec{u}_l \tag{32}$$

where $\left\{\vec{s}^m \in \mathbb{R}^D\right\}_{m=1}^{M}$ denotes manifold coordinates and s_0 denotes the manifold's center norm.
In this setup classification capacity is given by:

$$\alpha_c^{-1} = \int D^{D+1}\vec{t} \min_{S\vec{v}\geq 0} \|\vec{v} - \vec{t}\|^2 \tag{33}$$

where $S \in \mathbb{R}^{M\times(D+1)}$ denotes the manifold coordinates such that the $D+1$ coordinate of each row represents the manifold center:

$$S = \begin{bmatrix} \vec{s}^1 - \bar{s} & s_0 \\ \vdots & s_0 \\ \vec{s}^m - \bar{s} & s_0 \end{bmatrix} \tag{34}$$

$$\bar{s} = \frac{1}{M}\sum \vec{s}^m \tag{35}$$

$$s_0 = \|\bar{s}\| \tag{36}$$

Thus exact calculation of capacity requires to solve the following optimization problem:

$$\vec{v}\left(\vec{t}\right) = \arg\min_{S\vec{v}\geq 0} \|\vec{v} - \vec{t}\|^2 \tag{37}$$

with Lagrangian (written such that the $D+1$ coordinate is denoted v_0, t_0):

$$\mathcal{L} = \frac{1}{2}\sum_{l=0}^{D} (v_l - t_l)^2 - \sum^{M} \lambda_m \left(\sum s_l^m v_l - \sum \bar{s}_l v_l + s_0 v_0\right) \tag{38}$$

Then manifold properties are then defined in terms of the solution:

$$\tilde{s}_l = \frac{v_l - t_l}{v_0 - t_0} \tag{39}$$

$$R_M = \left[\sqrt{\sum_{l}^{D} \delta\tilde{s}_l^2}\right] \tag{40}$$

$$D_M = \left[\sum_{l}^{D} \frac{t_l \delta\tilde{s}_l}{\|\delta\tilde{s}\|}\right]^2 \tag{41}$$

Properties of random point-cloud manifolds We seek to analyze R_M and D_M for the case of random point-cloud manifolds, i.e. when $\{s_l^m \sim \mathcal{N}(0,1)\}_{m=1..M}^{l=0..D}$ and are sampled independently. When $D \gg M$ for a random $\{\vec{s}^m\}_{m=1}^{M}$ we further assume that $\vec{s}^{m_1} \perp \vec{s}^{m_2}$ for $m_1 \neq m_2$ and thus can simplify the analysis through an orthogonal change of base from D to the standard base $\vec{s}^m = \vec{e}^m$ of an M-dimensioal space. In this case we have $\bar{s} \equiv 1/M$ so that and $s_0 = 1/\sqrt{M}$:

$$S = \begin{bmatrix} \vec{e}^1 - 1/M & s_0 \\ \vdots & s_0 \\ \vec{e}^m - 1/M & s_0 \end{bmatrix} \tag{42}$$

so the Lagrangian becomes:

$$\mathcal{L} = \frac{1}{2}\sum_i^M (v_i - t_i)^2 + \frac{1}{2}(v_0 - t_0)^2 - \sum_m^M \lambda_m \left(v_m - \frac{1}{M}\sum_j^M v_j + s_0 v_0 \right) \tag{43}$$

An exact solution to this optimization problem is derived using Kuhn-Tucker conditions and is given by:

$$\lambda_l\left(\vec{t}\right) = \left[-t_l + \frac{1}{M}\sum_j^M t_j - \frac{t_0}{\sqrt{M}} \right]_+ \tag{44}$$

$$\bar{\lambda}\left(\vec{t}\right) = \frac{1}{M}\sum \lambda_l \tag{45}$$

$$v_l\left(\vec{t}\right) = t_l + \lambda_l - \bar{\lambda} \tag{46}$$

$$v_0\left(\vec{t}\right) = t_0 + \sqrt{M}\bar{\lambda} \tag{47}$$

Then the exact expression for $\delta\vec{s}$ is:

$$\delta\tilde{s}_l = \frac{v_l - t_l}{v_0 - t_0} = \frac{\lambda_l - \bar{\lambda}}{\bar{\lambda}\sqrt{M}} \tag{48}$$

By replacing the exact expression for λ from equation (44) with an approximation:

$$\lambda_l\left(\vec{t}\right) = [-t_l]_+ \tag{49}$$

we can analytically calculate the mean-field manifold properties:

$$D_M = \frac{\pi}{2(\pi - 1)} M \tag{50}$$

$$R_M = \sqrt{\pi - 1} \tag{51}$$

$$\alpha_c = \frac{2}{M} \tag{52}$$

References

[1] Kaiming He, Xiangyu Zhang, Shaoqing Ren, and Jian Sun. Deep Residual Learning for Image Recognition. *2016 IEEE Conference on Computer Vision and Pattern Recognition (CVPR)*, pages 770–778, 2016.

[2] SueYeon Chung, Daniel D. Lee, and Haim Sompolinsky. Classification and Geometry of General Perceptual Manifolds. *Physical Review X*, 8(3):031003, jul 2018.

[3] SueYeon Chung, Daniel D. Lee, and Haim Sompolinsky. Linear readout of object manifolds. *Physical Review E*, 93(6):060301, 2016.

[4] Zaiwen Wen and Wotao Yin. A feasible method for optimization with orthogonality constraints. *Mathematical Programming*, 142(1-2):397–434, 2013.

[5] SueYeon Chung, Uri Cohen, Haim Sompolinsky, and Daniel D. Lee. Learning Data Manifolds with a Cutting Plane Method. *Neural Computation*, 30(10):2593–2615, oct 2018.

[6] Elizabeth J. Gardner. The space of interactions in neural network models. *Journal of Physics A: Mathematical and General*, 21(1):257–270, 1988.
